# Processing of multi-digit additions in high math-anxious individuals: psychophysiological evidence

**DOI:** 10.3389/fpsyg.2015.01268

**Published:** 2015-08-21

**Authors:** María Isabel Núñez-Peña, Macarena Suárez-Pellicioni

**Affiliations:** ^1^Department of Behavioral Sciences Methods, Faculty of Psychology, University of BarcelonaBarcelona, Spain; ^2^Institute for Brain, Cognition, and Behavior (IR3C), University of BarcelonaBarcelona, Spain

**Keywords:** math anxiety, arithmetic processing, multi-digit additions, ERPs, P2, LPC

## Abstract

We investigated the time course of neural processing of multi-digit additions in high- (HMA) and low-math anxious (LMA) individuals. Seventeen HMA and 17 LMA individuals were presented with two-digit additions and were asked to perform a verification task. Behavioral data showed that HMA individuals were slower and more error prone than their LMA peers, and that incorrect solutions were solved more slowly and less accurately than correct ones. Moreover, HMA individuals tended to need more time and commit more errors when having to verify incorrect solutions than correct ones. ERPs time-locked to the presentation of the addends (calculation phase) and to the presentation of the proposed solution (verification phase) were also analyzed. In both phases, a P2 component of larger amplitude was found for HMA individuals than for their LMA peers. Because the P2 component is considered to be a biomarker of the mobilization of attentional resources toward emotionally negative stimuli, these results suggest that HMA individuals may have invested more attentional resources both when processing the addends (calculation phase) and when they had to report whether the proposed solution was correct or not (verification phase), as compared to their LMA peers. Moreover, in the verification phase, LMA individuals showed a larger late positive component (LPC) for incorrect solutions at parietal electrodes than their HMA counterparts. The smaller LPC shown by HMA individuals when verifying incorrect solutions suggests that these solutions may have been appeared more plausible to them than to their LMA counterparts.

## Introduction

In modern-day society, people are heavily dependent on technologies in both their professional and their everyday lives, so it is very important for them to be competent in the STEM (science, technology, engineering, and mathematics) fields. However, the technological advances of recent years have not been accompanied by a corresponding increase in students' mathematical abilities. In fact, a brief look at the latest PISA report (2012 Programme for International Student Assessment) confirms that 15-year old students from many of the Organisation for Economic Co-operation and Development (OECD) member countries have serious difficulties in mathematics (Organisation for Economic Co-operation and Development, 2013). One of the key factors related to math competence is math anxiety, because it has been demonstrated that high math-anxious individuals perform worse than their low math anxious peers on a wide range of numerical and mathematical tasks (Ashcraft et al., 2000; Ashcraft, 2002). Math anxiety is defined as a negative emotional response in situations involving mathematical reasoning that is characterized by avoidance as well as feelings of stress and anxiety (Ashcraft and Faust, 1994; Ashcraft and Ridley, 2005; see Suárez-Pellicioni et al., 2015 for a recent review). This avoidance of math-related situations limits math training in high math anxiety individuals, which in turn negatively affects their math performance and leads to lower grades. Later, when those school-aged children become adults, a low level of math proficiency will reduce their job opportunities and salary prospects (Bynner and Parsons, 1997). Because math anxiety is a problem in today's society, it merits in-depth investigation in order to increase our understanding of the factors contributing to its origin and maintenance.

To date, three accounts have been proposed to explain why high math-anxious individuals (henceforth, HMA) have a poorer performance in mathematics than their low math-anxious peers (henceforth, LMA). First, Ashcraft and colleagues (Ashcraft et al., 2000; Ashcraft and Kirk, 2001; Ashcraft and Krause, 2007) suggested that in HMA individuals a part of the working memory is occupied with worry and intrusive thoughts during performance of numerical task; as a result, they lack sufficient working memory resources to perform the task at hand and their performance deteriorates. The second proposal was formulated some years later by Maloney et al. (2010, 2011) who stated that HMA individuals may have a less precise representation of numerical magnitude, which would compromise the development of more complex math skills. Finally, the third proposal is by Suárez-Pellicioni et al. (2013, 2014), who suggested that HMA individuals have an attentional control deficit which makes them more susceptible to distraction in numerical tasks.

The attentional control theory (Eysenck et al., 2007) explains the relationship between emotion, attention and cognitive performance. This theory proposes that “anxiety affects performance via its adverse effects on attentional control” (Eysenck et al., 2007, p. 170), which is a key function of the central executive component of the working memory (Baddeley, 1986). Specifically, anxiety affects the efficiency of the inhibition function (which uses attentional control to prevent attention being directed to task-irrelevant stimuli and responses) and the shifting function (which uses attentional control in a positive way to respond optimally to changing task requirements). As a consequence, high anxious individuals need to increase the recruitment of any available attentional resources in order to perform the task at hand.

The effects of emotion on attention have been studied in the general population and in both clinical (i.e., anxiety and depression disorders) and non-clinical individuals reporting high levels of anxiety (for a review, see Yiend, 2010). It has been found that emotional material matching individuals' emotional characteristics is attended differently than non-emotional material, and that this effect is similar in clinically anxious and non-clinical high-anxious individuals (Bar-Haim et al., 2007). It has been suggested that the attentional system of anxious individuals may be abnormally sensitive to threat-related stimuli in the environment (Bar-Haim et al., 2005).

Cognitive neuroscientists have used the recording of brain activity at the scalp by means of the event-related brain potential (ERP) technique to study the interaction between attentional and emotional processes (for a review see Hajcak et al., 2010, 2012). ERPs allow for a more direct assessment of these processes than behavioral measures because they obtain an online measure of attentional processing of emotional information. One commonly used component in the study of the effects of emotion on attention is P2, a positive peak with a latency at 200 ms following stimulus onset which is elicited by emotionally negative stimuli (Carretié et al., 2001, 2004). Studies of the P2 component in the clinical population have found that high-anxious participants have greater P2 amplitudes than low-anxious participants when presented with angry faces (Bar-Haim et al., 2005; Eldar et al., 2010). P2 enhancement was also found when less beautiful pendants were presented to a non-clinical population as compared to more beautiful ones (Wang et al., 2012). The increase in P2 amplitude elicited by negative events has been suggested to be a reflection of the mobilization of attentional resources toward emotionally negative stimuli (Carretié et al., 2001; Wang et al., 2012).

In the present study, ERPs were recorded while HMA and LMA individuals performed a multi-digit verification task. This difficult arithmetic task was selected because, according to the attentional control theory (Eysenck et al., 2007), anxiety impairs attentional control, especially during heightened states of anxiety when overall task processing demands are high. Although previous studies have explored single-digit addition solving in LMA and HMA individuals (Suárez-Pellicioni et al., 2013), to the best of our knowledge, no study to date has explored the psychophysiological correlate of more complex addition problem solving in these individuals. Moreover, while in Suárez-Pellicioni et al. (2013) we centered only in the verification of additions, in this study we explored addition solving in a more complete way, by examining early brain activity differences between high- and low-math anxious individuals in both the calculation and verification phases of the arithmetic task. Furthermore, given previous evidence suggesting differences in processing incorrect proposed solutions for simple additions in HMA individuals (Suárez-Pellicioni et al., 2013), the second objective of this study was to examine the verification phase in more depth in order to explore possible group differences when incorrect solutions are presented for a more complex addition task.

As regards behavioral measures, we expected slower response times and lower hit rates in HMA individuals than in their LMA counterparts, because it has been demonstrated that as arithmetic tasks become more complex, the negative effects of anxiety on performance are more evident (Ashcraft and Faust, 1994; Faust et al., 1996). Moreover, we expected incorrect solutions to be solved more slowly and less accurately than correct ones since both solutions showed the same unit number and for incorrect solutions the ten was always one point above the ten in the correct solution (e.g., 27 + 16 = 53). Thus, incorrect solutions were plausible solutions to the addition and were expected to be more difficult to verify than the correct ones (El Yagoubi et al., 2003; Núñez-Peña and Escera, 2007). Finally, we expected that the incorrect solutions would appear to be more plausible solutions to HMA than to LMA individuals, given that HMA are expected to have more difficulties with math and to have committed more errors of this type when solving these additions.

As for ERP data, we expected to find different patterns of neural activity in the two groups. Specifically, we expected the multi-digit addition task to mobilize more attentional resources in HMA individuals than in LMA during both the calculation and the verification phases, because multi-digit additions would be emotionally negative stimuli for HMA individuals. As a consequence, we expected a larger P2 component in HMA individuals than in their LMA peers. In addition to these early ERP differences between groups, we also expected differences in the late positive component (LPC) in the verification phase. Previous studies have shown that a LPC with latency around 500 ms post-stimulus and with parietal distribution is elicited whenever an incorrect solution is presented in an arithmetic verification task (Niedeggen et al., 1999; Szucs and Soltész, 2010; Núñez-Peña and Suárez-Pellicioni, 2012). More importantly, differences in the amplitude of this positive component according to arithmetical ability have been found when an incorrect solution very close to the correct one is presented (i.e., a plausible solution such as 9 in the addition 3+5), with lower-skilled arithmetic problem-solvers showing a smaller LPC than their higher-skilled counterparts (Núñez-Peña and Suárez-Pellicioni, 2012). This result has been interpreted as an indication of differences in the strength of association between problems and potential solutions depending on arithmetical skills. More specifically, low-skilled individuals are considered to use an exhaustive verification strategy when presented with a plausible incorrect solution because they have been more frequently exposed to this type of error. Thus, the LPC is believed to be a measure of the degree of expectancy or plausibility of the solution presented. In this study, incorrect solutions were expected to elicit a larger LPC in LMA than in HMA individuals, because the latter group, considered to have more difficulties with math, might have higher strength of association between problems and this type of incorrect solutions in their memory, perceiving these incorrect solutions as more plausible than their LMA peers.

## Materials and methods

### Participants

Thirty-four healthy volunteers were tested in this study, half of them with a high level of math anxiety (HMA) and the other half with a low level (LMA). Participants were selected from a sample of 629 students of the degree in Psychology at the University of Barcelona who were assessed for math anxiety, trait anxiety and math ability (see Materials and Methods).

The LMA group comprised 17 participants (age range = 19–31, mean = 22.06, standard deviation = 3.54, 16 right-handed) who scored below the first quartile in the Shortened Mathematics Anxiety Rating Scale (sMARS) (Alexander and Martray, 1989) (score range = 30–56, mean = 43.94, standard deviation = 7.36). The HMA group comprised 17 participants (age range = 19–31, mean = 21.94, standard deviation = 2.98, 16 right-handed) who scored above the third quartile in the sMARS (score range = 81–99, mean = 87.41, standard deviation = 5.17).

Groups differed in math anxiety [*t*_(32)_ = 19.92, *p* < 0.001], but not in trait anxiety [*t*_(32)_ = 0.87, *p* = 0.39], age [*t*_(32)_ = 0.10, *p* = 0.91], years of formal education [*t*_(32)_ = 1.13, *p* = 0.26], or handedness (χ^2^ = 0.00, *p* = 1). Differences between groups were found in math ability, showing that HMA individuals correctly solved less additions (mean = 0.16, standard deviation = 0.04) than their LMA peers (mean = 0.21, standard deviation = 0.07) [*t*_(32)_ = 2.01, *p* = 0.05].

All had normal or corrected-to-normal visual acuity and none reported any history of neurological or psychiatric disorders. Participants were naïve as to the purposes of the study and gave written informed consent before the experiment. The experimental protocol was approved by the Ethics Committee of the University of Barcelona and was in accordance with the Code of Ethics of the World Medical Association (Declaration of Helsinki).

### Material

#### Shortened mathematics anxiety rating scale (sMARS) (Alexander and Martray, 1989)

The sMARS is a 25-item version of the Math Anxiety Rating Scale (MARS) (Richardson and Suinn, 1972). This instrument measures anxiety by presenting 25 situations which may cause math anxiety (e.g., *Being given a homework assignment with many difficult problems that are due in the next class meeting*). The participant decides on the level of anxiety associated with the item by providing a score on a five-point Likert scale ranging from 1 (no anxiety) to 5 (high anxiety). The sum of the item scores provides the total score for the instrument, which ranges from 25 to 125. In the present study, we used the Spanish version of the sMARS (Núñez-Peña et al., 2013). The scores for the Spanish version of the sMARS have shown strong internal consistency (Cronbach's alpha = 0.94) and high 7-week test-retest reliability (intra-class correlation coefficient = 0.72).

#### State-trait anxiety inventory (STAI) (Spielberger et al., 1983)

The STAI is a 40-item scale used to measure state (STAI-S) and trait (STAI-T) anxiety. Only the score on the STAI-T was considered in this study, since it reflects a more general and relatively stable tendency to respond with anxiety. This inventory includes 20 statements describing different emotions, and participants answer by considering how they feel “in general.” Items are answered on a four-point Likert scale, from 0 (almost never) to 3 (almost always). Good to excellent internal consistency (Cronbach's alpha = 0.95), adequate 30-day test-retest reliability with high school students (*r* = 0.75) and 20-day test-retest reliability with college students (*r* = 0.86) has been reported for the Spanish version of this subscale (Spielberger et al., 2008).

#### Addition test from the french kit (French et al., 1963)

The first page of this test was used to assess participants' math ability. It consists of 60 additions involving three numbers of either one or two digits (e.g., 6 + 67 + 38), vertically presented. Participants were asked to solve the additions as fast and as accurately as possible during a 2-min period. The number of correctly solved additions over the total of additions presented in the test (i.e., 60) was taken as a measure of participants' arithmetical ability.

#### Calculation task

A two-digit addition task was presented to each participant during the recording session. Addends were comprised between 12 and 29 and were presented horizontally on the screen (e.g., 13 + 24). Addends were followed by the proposed solution, which could be correct (e.g., 37) or incorrect (+10 above the correct solution; e.g., 47). From all the possible combinations between the addends mentioned, the ones that could generate confusion with other processes (e.g., rule application) were discarded. More specifically, the numbers 20 and 21 (as well as 10 and 11), tie problems (e.g., 12 + 12) and consecutive addends (e.g., 12 + 13) were not included. From all the remaining possible combinations, 200 additions were randomly selected (the Appendix includes the 100 additions in their smaller + bigger number version). Numbers were presented in font size 50 (Courier New) in white against a black background and at subtended visual angles of 6.30° (addition) or 2.29° (proposed solution), horizontally and 1.48°, vertically.

### Procedure

Participants were tested individually. Upon entering the experimental room, participants completed standard procedures for informed consent and a demographics questionnaire asking about their age and number of years of formal education. EEG/EOG sensor electrodes were then attached and participants were given detailed task instructions. Next, participants were seated 100 cm away from the computer screen in an electrically-shielded, sound-attenuating recording chamber. The experimental session began with a training period of 25 trials, on which participants received feedback regarding their performance. The training trials were only used to familiarize the participants with the task and were excluded from the statistical analysis.

The participants' task consisted of indicating whether the proposed solution for the addition displayed was true or false by pressing the left or right button of the mouse. Response buttons were counterbalanced between subjects within each group. Participants were encouraged to answer as fast and as accurately as possible. Each trial began with a fixation sign (an asterisk) shown for 500 ms, which was followed by the addition, presented for 1500 ms with a pre- and post- 100 ms ISI. After this, the proposed solution appeared and remained on the screen until the participant gave a response (or for a maximum of 2000 ms), and then a 500 ms pause was shown. Finally, the trial ended with a variable inter-trial interval ranging from 600 to 900 ms (all pauses consisting of a black screen). The recording session consisted of four blocks of 50 stimuli (200 total trials) separated by a 1-min rest. Trials were randomly presented to each participant. The experiment, including electrode placement and execution of the practice and test phases, lasted about 120 min.

The E-prime 2.0 program (Psychology Software Tools Inc., Sharpsburg, PA, USA) was used to control the presentation and timing of the stimuli and the measurement of response accuracy and response times.

### Electrophysiological recording

The EEG was recorded with ANT hardware and software (B.V., Enschede, The Netherlands) from 64 electrodes positioned according to the extended 10/20 system, as well as two electrodes on the right and left mastoids, and mounted in a commercial WaveGuard EEG Cap (Eemagine Medical Imaging Solutions GmbH. ANT Advanced Neuro Technology). EEG channels were continuously digitized at a rate of 512 Hz by an ANT amplifier (B.V., Enschede, The Netherlands). A band-pass filter was set from 0.16 to 30 Hz, and electrode impedance was kept below 5 kΩ. The horizontal and vertical electrooculogram was recorded with electrodes placed at the outer canthus and below the right eye respectively. The common reference electrode was placed on the tip of the nose, and ground was located at AFz.

### Data analysis

Mean response times (RTs) for correctly solved trials and percentage of hits were calculated for each participant in each condition (correct and incorrect proposed solutions). RTs were calculated after removing trials with values exceeding 2.5 SD from participants' mean scores (outliers) (Van Selst and Jolicoeur, 1994).

Response time and the percentage of hits were analyzed with analyses of variance (ANOVAs) taking *Proposed Solution* (correct, incorrect) as the within-subject factors and *Group* (LMA, HMA) as the between-subjects factor. The *F*-value, the degrees of freedom, the probability level, and the $\eta_{p}^{2}$ effect size index are given. Whenever an interaction reached significance, simple effect analyses were performed and the Hochberg approach was used to control for the increase in Type I error (Keselman, 1998). Only significant effects (*p* ≤ 0.05) are reported.

ERP data time-locked both to the presentation of the two addends (henceforth, the calculation phase) and to the presentation of the proposed solution (henceforth, the verification phase) were then analyzed. Three ERP averages were calculated per participant: one for the calculation phase and two for the verification phase (for correct and incorrect solutions). The averages were constructed from −200 to 800 ms epochs relative to stimulus onset. Trials with voltages exceeding 20 standard deviations in the EOG electrodes and ±100 μV in the remaining electrodes were excluded from the ERP average. Ocular artifacts were identified and corrected with the eye-movement correction algorithm used in the EEprobe program (ANT, The Netherlands).

For the ERP analysis of the calculation phase, ANOVAs were performed on the ERP mean amplitudes in the 175–225 ms window in order to study the P2 component. Analysis was performed at nine electrodes (F3, Fz, F4, C3, Cz, C4, P3, Pz, P4), taking *Frontality* (frontal, central, parietal), and *Laterality* (three levels from left to right) as within-subject factors and *Group* (LMA, HMA) as the between-subjects factor. For the ERP analysis of the verification phase, ANOVAs were performed on the ERP mean amplitudes in the 175–225 ms window in order to study the P2 component and in the 400–600 ms window in order to study the LPC. The *F*-value, the uncorrected degrees of freedom, the probability level following correction, the ε-value (when appropriate), and the $\eta_{p}^{2}$ effect size index are given. Whenever an interaction reached significance, simple effect analyses were performed and the Hochberg approach was used to control for the increase in Type I error (Keselman, 1998). Only significant effects (*p* ≤ 0.05) are reported.

## Results

### Behavioral results

As far as response time was concerned, the main effects of Proposed solution [*F*_(1, 32)_ = 48.44, *p* < 0.001, $\eta_{p}^{2}=0.60$] and Group [*F*_(1, 32)_ = 6.36, *p* = 0.01, $\eta_{p}^{2}=0.16$] reached statistical significance. Incorrect proposed solution were verified more slowly (mean = 766.55 ms, SEM = 35.48 ms) than correct proposed solutions (mean = 694.32 ms, SEM = 32.44 ms), and HMA individuals were slower (mean = 815.20 ms, SEM = 47.51 ms) than their LMA peers (mean = 645.67 ms, SEM = 47.51 ms). More interestingly, the Proposed solution x Group interaction was marginally significant [*F*_(1.32)_ = 3.69, *p* = 0.06, $\eta_{p}^{2}=0.10$]. This interaction showed that the difference between incorrect and correct solutions was larger for the HMA (mean = 92.2 ms, SEM = 17.5) than for the LMA (mean = 52.3 ms, SEM = 11.1) group, [*t*_(32)_ = 1.92, *p* = 0.06]. Means and standard errors of response times for correct and incorrect proposed solutions for the LMA and HMA groups are given in Table 1.

**Table 1:** **Means of response times (in ms) and of percentage of hits (standard error of the mean in brackets) for correct and incorrect proposed solutions for the LMA and HMA groups**.

	**Response times**		**Percentage of hits**	
	**Incorrect**	**Correct**	**Incorrect**	**Correct**
LMA	671.82 (41.86)	619.53 (40.23)	88.29 (2.69)	92.68 (1.63)
HMA	861.29 (57.30)	769.12 (50.90)	79.52 (2.69)	85.06 (1.63)

*LMA, Low math-anxious group; HMA, High math-anxious group*.

As for the percentage of hits, the main effects of Proposed solution [*F*_(1, 32)_ = 18.12, *p* < 0.001, $\eta_{p}^{2}=0.36$] and Group [*F*_(1, 32)_ = 7.84, *p* = 0.009, $\eta_{p}^{2}=0.19$] reached statistical significance. Incorrect proposed solutions were verified less accurately (mean = 83.9, SEM = 1.9) than correct proposed solutions (mean = 88.9, SEM = 1.1), and HMA individuals made fewer hits (mean = 82.3, SEM = 2.1) than their LMA peers (mean = 90.4, SEM = 2.1). The analysis of the differences in percentage of hits between correct and incorrect solutions revealed a marginally significant effect of Group [*F*_(1, 32)_ = 3.455, *p* = 0.07, $\eta_{p}^{2}=0.10$], showing that the decrease in percentage of hits from correct to incorrect solutions was larger in the HMA group (mean = −12.9, SEM = 2.3) than in the LMA group (mean = −6.9, SEM = 2.3). Means and standard errors of percentage of hits for correct and incorrect proposed solutions for the LMA and HMA groups are given in Table 1.

### ERP results: Calculation phase

**Figure 1:**
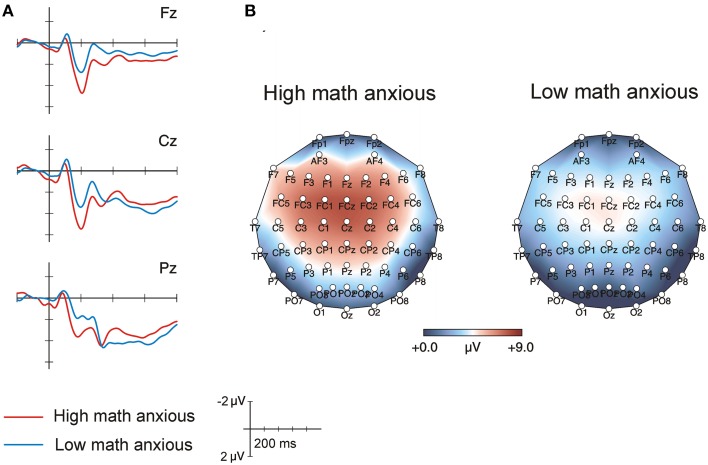
**(A)** Raw averaged waveforms for HMA (*n* = 17) and LMA (*n* = 17) individuals during the calculation phase. **(B)** Topographic maps waveforms for HMA and LMA individuals in the 175–225 ms window of the calculation phase.

Figure 1A shows the grand-average ERPs for each group in the calculation phase at frontal, central and parietal electrodes. The differences between groups were evident at about 200 ms post-stimulus, where HMA individuals showed a larger P2 component than their LMA peers. Scalp topographic maps in Figure 1B reveal brain activity in the 175–225 ms window for both groups; they show that the positive component was larger in the HMA group than in the LMA group and that this P2 component was widely distributed. Topographic maps were plotted using the EEProbe 3.1 program (ANT Software BV, Enschede, The Netherlands).

The statistical analysis performed on the 175–225 ms window supports these observations. The overall ANOVA revealed a significant effect of Group [*F*_(1, 32)_ = 6.72, *p* = 0.014, $\eta_{p}^{2}=0.17$] showing that voltage was more positive for HMA individuals (mean = 5.9, SEM = 0.6) than for their LMA counterparts (mean = 3.5, SEM = 0.6). None of the interactions with the Group factor reached significance. Table 2 shows amplitude means and standard errors for both groups in all the electrodes analyzed.

**Table 2:** **Mean amplitudes (in μV) and standard error (in brackets) for the P2 component in the calculation phase (175–225 ms) for the HMA and LMA groups**.

	**F3**	**Fz**	**F4**	**C3**	**Cz**	**C4**	**P3**	**Pz**	**P4**
HMA	6.28 (0.54)	6.61 (0.57)	6.17 (0.54)	6.91 (0.72)	7.78 (0.84)	6.27 (0.78)	4.34 (0.76)	5.09 (0.94)	4.18 (0.90)
LMA	3.94 (0.54)	3.90 (0.57)	3.91 (0.54)	4.27 (0.72)	4.81 (0.84)	3.73 (0.78)	2.27 (0.76)	2.65 (0.94)	2.10 (0.90)

### ERP results: Verification phase

**Figure 2:**
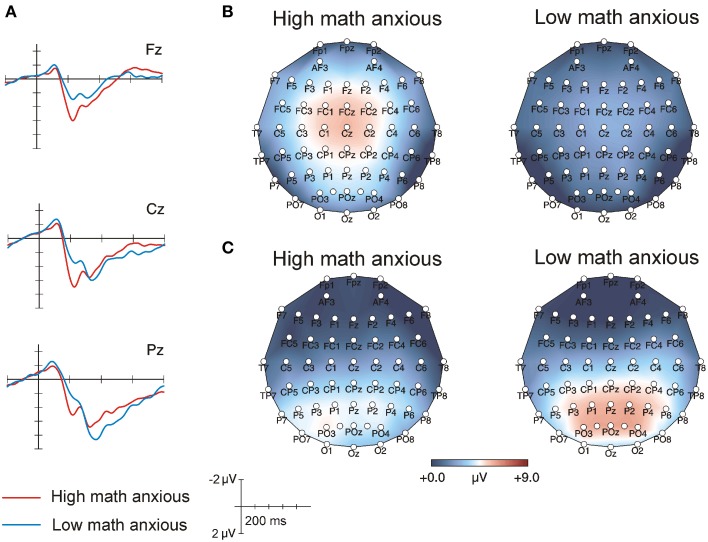
**(A)** Raw averaged waveforms for HMA (*n* = 17) and LMA (*n* = 17) individuals during the verification phase when correct solutions were presented. **(B)** Topographic maps for HMA and LMA individuals in the 175–225 ms window of the verification phase. **(C)** Topographic maps for HMA and LMA individuals in the 400–600 ms window of the verification phase.

Figure 2A shows the grand-average ERPs for each group in the verification phase for correct proposed solutions at frontal, central and parietal electrodes. The differences between groups were evident at about 200 ms post-stimulus, when HMA individuals showed a larger P2 component than their LMA peers. Differences between groups were also evident later, when the LMA group showed a larger LPC, peaking about 400 ms post-stimulus compared with their HMA counterparts. This effect was larger at parietal positions. Scalp topographic maps in Figures 2B,C show brain activity in the 175–225 and the 400–600 ms windows for both groups. Figure 2B reveals that the P2 component was frontocentrally distributed and was larger in the HMA group than in LMA. Figure 2C shows that the LPC was parietally distributed and was larger in the LMA group than in HMA.

**Figure 3:**
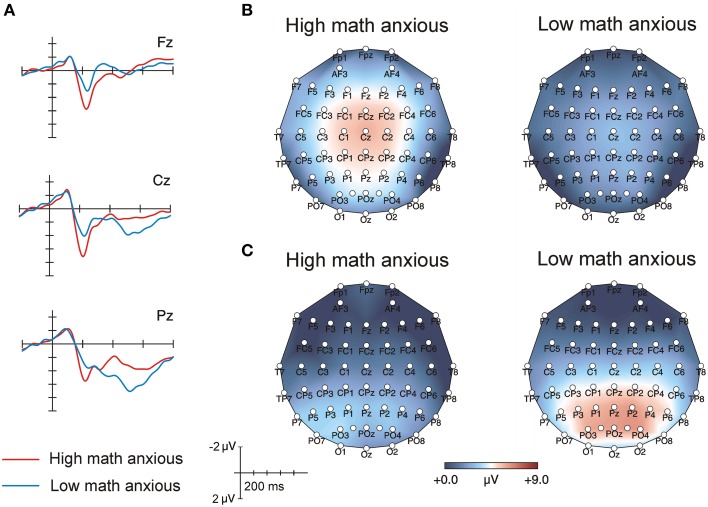
**(A)** Raw averaged waveforms for HMA (*n* = 17) and LMA (*n* = 17) individuals during the verification phase when incorrect solutions were presented. **(B)** Topographic maps for HMA and LMA individuals in the 175–225 ms window of the verification phase. **(C)** Topographic maps for HMA and LMA individuals in the 400–600 ms window of the verification phase.

Figure 3A shows the grand-average ERPs for each group in the verification phase for incorrect proposed solutions at frontal, central and parietal electrodes. Figures 3B,C showed scalp topographic maps for the P2 and the LPC for both groups in incorrect proposed solutions. For these components, these figures showed ERP patterns similar to the ones described above for correct solutions.

The ANOVA performed on the 175–225 ms window revealed a significant main effect of Group [*F*_(1, 32)_ = 15.01, *p* < 0.001, $\eta_{p}^{2}=0.32$], showing that voltage was more positive for HMA individuals (mean = 4.2, SEM = 0.4) than for their LMA counterparts (mean = 1.9, SEM = 0.4). None of the interactions with the Group factor reached statistical significance. Moreover, neither the main effect of Proposed solution nor any of the interactions with this factor were significant. Table 3 shows amplitude means and standard errors for both groups in all the electrodes analyzed.

**Table 3:** **Mean amplitudes (in μV) and standard error (in brackets) for the P2 component in the verification phase (175–225 ms) for the HMA and LMA groups**.

	**F3**	**Fz**	**F4**	**C3**	**Cz**	**C4**	**P3**	**Pz**	**P4**
HMA	3.64 (0.39)	4.42 (0.41)	3.78 (0.43)	4.49 (0.51)	5.80 (0.52)	4.11 (0.50)	3.90 (0.54)	4.53 (0.59)	3.58 (0.53)
LMA	1.39 (0.39)	1.85 (0.41)	1.59 (0.43)	1.87 (0.51)	2.84 (0.52)	2.13 (0.50)	1.55 (0.54)	2.18 (0.59)	1.68 (0.53)

As for the ANOVA performed on the 400–600 ms window, the interactions Group × Frontality [*F*_(2, 64)_ = 4.98, *p* = 0.01, ε = 0.14, $\eta_{p}^{2}=0.22$] and Proposed solution × Frontality, [*F*_(1, 64)_ = 3.54, *p* = 0.04, ε = 0.69, $\eta_{p}^{2}=0.10$] were statistically significant. Separate follow-up ANOVAs were computed for each level of frontality, showing that the Group effect [*F*_(1, 32)_ = 3.69, *p* = 0.06, $\eta_{p}^{2}=0.10$] and the Group x Proposed solution interaction were marginally significant at parietal positions [*F*_(1, 32)_ = 3.43, *p* = 0.07, $\eta_{p}^{2}=0.09$]. In order to study this interaction in more detail, simple effects analysis was performed and results showed that amplitude was more positive for LMA individuals (mean = 5.6, SEM = 0.8) than for their HMA peers (mean = 3.1, SEM = 0.8) at parietal sites for incorrect solutions [*F*_(1, 32)_ = 4.9, *p* = 0.03, $\eta_{p}^{2}=0.13$] but not for the correct ones, which showed no differences between groups. No significant effects were found at frontal and central positions. Table 4 shows amplitude means and standard errors for both groups for correct and incorrect solutions in all the electrodes analyzed.

**Table 4:** **Mean amplitudes (in μV) and standard error (in brackets) for the LPC component in the verification phase (400–600 ms) for the HMA and LMA groups**.

	**F3**	**Fz**	**F4**	**C3**	**Cz**	**C4**	**P3**	**Pz**	**P4**
**CORRECT SOLUTIONS**									
HMA	0.03 (0.66)	0.22 (0.66)	0.27 (0.70)	1.26 (0.72)	1.89 (0.87)	2.29 (0.71)	4.33 (0.67)	4.22 (0.74)	4.05 (0.67)
LMA	−0.18 (0.66)	−0.27 (0.66)	0.22 (0.70)	2.48 (0.72)	2.74 (0.87)	3.44 (0.71)	5.23 (0.67)	5.74 (0.74)	5.41 (0.67)
**INCORRECT SOLUTIONS**									
HMA	−0.02 (0.80)	0.17 (0.83)	−0.19 (0.85)	0.71 (0.91)	1.34 (1.04)	1.34 (0.88)	3.34 (0.85)	3.06 (0.91)	2.84 (0.78)
LMA	0.32 (0.80)	0.09 (0.83)	0.07 (0.85)	2.74 (0.91)	2.94 (1.04)	3.27 (0.88)	5.32 (0.85)	6.04 (0.91)	5.57 (0.78)

## Discussion

The present study examined the behavioral and electrophysiological measures of HMA and LMA individuals when performing a multi-digit addition verification task, by focusing on both the calculation and the verification phases. To our knowledge this is the first time that multi-digit addition processing has been studied in HMA individuals by means of the ERP technique. Our objective was two-fold. First, we were interested in studying attentional processes. According to the attentional control theory (Eysenck et al., 2007), we expected that HMA individuals would need to allocate more attentional resources to perform the arithmetical task than their LMA counterparts, because they would perceive multi-digit additions as emotionally negative stimuli. Therefore, we expected larger P2 amplitudes in math-anxious participants. Second, we aimed to study differences in the processing of incorrect solutions between the two groups. Specifically, we expected to find between-group differences in the amplitude of the LPC, a component whose amplitude increases with the implausibility of the solution presented in an arithmetic verification task (Niedeggen et al., 1999; Szucs and Soltész, 2010; Núñez-Peña and Suárez-Pellicioni, 2012). In the present study, incorrect solutions were expected to appear more plausible to HMA than to LMA individuals, because the former would have committed more errors of this type, so the association between the addition and the incorrect solution would be stronger for them. Therefore, using ERP methodology, we expected LMA individuals to show a larger LPC for incorrect proposed solutions than their HMA peers.

The behavioral results of the study partially confirmed our predictions. Regarding behavioral measures, HMA individuals were slower and more error prone than their LMA counterparts. This result was expected because math anxiety and math competence have shown a consistent, negative relationship (Ashcraft and Ridley, 2005). Moreover, correct solutions were solved faster and more accurately than incorrect ones. This result was also expected because incorrect solutions were constructed by adding 10 to the correct solution, being, therefore, plausible solutions requiring participants to check the tens. Finally, as for the analyses of the difference scores in percentage of hits and response time, we found that the decrease in percentage of hits and increase in response times from correct to incorrect solutions tended to be larger in the HMA group than in the LMA group. This result suggests that as we had predicted, incorrect solutions seemed to be more plausible to the HMA group as compared to their LMA peers. However, differences between groups were marginally significant (*p*-values of 0.06 and 0.07 for response times and hit rates, respectively) and effect sizes were low ($\eta_{p}^{2}$ of 0.1 for both measures), so these effects need further investigation.

With regard to electrophysiological measures, our data revealed two relevant results. First, between-group differences were found at about 200 ms post-stimulus in both the calculation and the verification phases of the arithmetic task. Specifically, HMA individuals presented greater P2 amplitude than their LMA peers. Because increased P2 amplitude is elicited in response to stimuli arousing negative feelings and is considered to be an indicator of the mobilization of attentional resources to the stimulus processing (Carretié et al., 2001, 2004), the present findings suggest that HMA individuals needed to invest more attentional recourses to perform the arithmetic task than their LMA peers. Moreover, the fact that this effect was present in both the calculation and verification phases provides evidence that it is a very robust effect and that the stronger mobilization of attentional resources in HMA individuals was needed not only in the initial step of the calculation process (when both addends are presented in the calculation phase) but also in the final step of the verification process (when the proposed solution is presented). However, although HMA individuals invested more attentional resources, they still needed more time and committed more errors when solving the verification task. In line with the attentional control theory (Eysenck et al., 2007), this finding suggests that HMA individuals not only showed differences in what Eysenck et al. called *performance effectiveness*, that is, in their level of performance on the task (behavioral measures), but also in *processing efficiency*, given that using more resources (P2 amplitude) to achieve their level of performance made their processing less efficient.

The second psychophysiological result in the present study is that between-group differences were also found for incorrect proposed solutions in the verification phase. These differences emerged at a late stage of processing: a LPC with latency about 500 ms post-stimulus showed a larger amplitude for LMA than for HMA individuals at parietal electrodes. Previous research has shown that whenever an incorrect solution is presented in an arithmetic verification task, a parietal LPC is elicited and its amplitude is modulated by the distance between the correct and the incorrect proposed solution (Niedeggen et al., 1999; Szucs and Csépe, 2004, 2005; Núñez-Peña and Escera, 2007; Szucs and Soltész, 2010). In addition, Núñez-Peña and Suárez-Pellicioni (2012) found a modulation in the LPC amplitude depending on arithmetical ability, reporting a smaller LPC for lower-skilled individuals. Since LPC amplitude is taken as an indicator of the plausibility of the solution, they suggested that incorrect solutions close to the correct ones appear to be more plausible to low-skilled individuals than to their high-skilled peers.

Our result concerning the LPC differences between groups can be explained in terms of the degree of plausibility of the proposed incorrect solution for high- and low-math anxious individuals. In our study, we created incorrect solutions by adding 10 to the correct solution, so that participants needed to calculate the tens in order to correctly verify the additions. In this way, our incorrect solutions were plausible solutions for the addition at hand. The fact that a reduction in the amplitude of this component was shown in HMA individuals for incorrect solutions suggests that these solutions were more plausible to them than to their LMA counterparts. This difference may be due to the fact that, in line with the well-known negative correlation between math anxiety and math competence (Hembree, 1990), HMA individuals would have been less skilled than their LMA peers. The present interpretation of this result is also in accordance with our behavioral results, which showed that the increase in RTs and the decrease in hit rates for incorrect solutions compared with the correct ones tended to be larger for HMA individuals.

As a whole, one of the main implications of this study is that it is the first one finding an enhanced P2 component in math anxiety, suggesting that numbers may have generated an emotionally negative response in HMA individuals, in the same way as other stimuli generated the same response in other anxious populations (e.g., angry faces; Eldar et al., 2010). Moreover, the results of this study can better be explained by one of the three accounts that explain the negative effects of math anxiety on performance. In line with Ashcraft and colleagues' account (Ashcraft et al., 2000; Ashcraft and Kirk, 2001; Ashcraft and Krause, 2007), HMA individuals would have experienced intrusive thoughts regarding the math task (e.g., doubts about being able to perform well, etc.) so, in order to compensate for this detrimental effect of math anxiety, HMA individuals may have increased their attentional resources (cognitive effort), which would have been reflected in the increase on the P2 component. However, even with this effort, they still performed worse (were slower and made fewer hits) than their LMA peers.

In conclusion, this study has been the first in demonstrating that HMA individuals show larger amplitudes of the attention-related P2 ERP component than their LMA counterparts when performing a two-digit addition verification task. This is a very robust effect because P2 differences between groups were found when both addends were presented (the calculation phase) and also when the proposed solution was presented (the verification phase). These findings may suggest that a complex arithmetic task elicited greater mobilization of attentional resources in HMA than in LMA individuals, probably because such a numeric task elicited a negative emotional response in those individuals high in anxiety toward math. Moreover, the larger LPC amplitude found for HMA individuals in incorrect proposed solutions might indicate that this type of solution appears to be more plausible to them than to their LMA peers, due to the fact that they would have committed more errors of this type when solving additions. Despite the relevant results raised by this study, a limitation should be acknowledged that has to do with the natural relationship between math anxiety and math ability, an association that posits difficulties in order to study math anxiety, given that the effects of math ability would always be, somehow, intervening in explaining the effects found.

## Funding

This research was supported by the Spanish Ministry of Economy and Competitiveness under grant PSI2012-35703, the Spanish Ministry of Science and Technology under grant BES-2010-036859, and the Generalitat de Catalunya under grant SGR2014-177.

### Conflict of interest statement

The authors declare that the research was conducted in the absence of any commercial or financial relationships that could be construed as a potential conflict of interest.

## Appendix

**Table:**

**Addition**	**Correct**	**Incorrect**	**Addition**	**Correct**	**Incorrect**	**Addition**	**Correct**	**Incorrect**
12 + 14	26	36	16 + 23	39	49	24 + 16	40	50
12 + 17	29	39	16 + 25	41	51	24 + 19	43	53
12 + 19	31	41	16 + 29	45	55	24 + 22	46	56
12 + 23	35	45	17 + 12	29	39	24 + 23	47	57
12 + 25	37	47	17 + 14	31	41	24 + 28	52	62
12 + 27	39	49	17 + 22	39	49	25 + 12	37	47
13 + 12	25	35	17 + 25	42	52	25 + 13	38	48
13 + 15	28	38	17 + 26	43	53	25 + 14	39	49
13 + 19	32	42	17 + 29	46	56	25 + 16	41	51
13 + 22	35	45	18 + 14	32	42	25 + 19	44	54
13 + 24	37	47	18 + 23	41	51	25 + 22	47	57
13 + 26	39	49	18 + 25	43	53	25 + 28	53	63
13 + 27	40	50	18 + 29	47	57	26 + 12	38	48
13 + 29	42	52	19 + 1 2	31	41	26 + 13	39	49
14 + 12	26	36	19 + 23	42	52	26 + 15	41	51
14 + 17	31	41	19 + 29	48	58	26 + 19	45	55
14 + 19	33	43	22 + 12	34	44	26 + 22	48	58
14 + 22	36	46	22 + 14	36	46	26 + 23	49	59
14 + 23	37	47	22 + 17	39	49	26 + 28	54	64
14 + 24	38	48	22 + 18	40	50	27 + 12	39	49
14 + 25	39	49	22 + 19	41	51	27 + 13	40	50
14 + 28	42	52	22 + 24	46	56	27 + 16	43	53
15 + 12	27	37	22 + 27	49	59	27 + 18	45	55
15 + 13	28	38	22 + 29	51	61	27 + 22	49	59
15 + 14	29	39	23 + 12	35	45	28 + 12	40	50
15 + 17	32	42	23 + 14	37	47	28 + 15	43	53
15 + 22	37	47	23 + 16	39	49	28 + 18	46	56
15 + 24	39	49	23 + 18	41	51	28 + 26	54	64
15 + 26	41	51	23 + 19	42	52	29 + 12	41	51
15 + 29	44	54	23 + 22	45	55	29 + 15	44	54
16 + 12	28	38	23 + 26	49	59	29 + 23	52	62
16 + 13	29	39	23 + 29	52	62	29 + 27	56	66
16 + 18	34	44	24 + 12	36	46			
16 + 22	38	48	24 + 15	39	49			
